# A Comparison Study of Doctor-Patient Internet Interactions in Traditional and Modern Medicine: Empirical Evidence from Online Healthcare Communities

**DOI:** 10.1155/2022/4619914

**Published:** 2022-10-07

**Authors:** Song Cao, Xiang Gao, Shuzhen Niu, Qian Wei

**Affiliations:** ^1^Nanyang Technological University, No. 50 Nanyang Avenue, Singapore 639798, Singapore; ^2^Research Center of Finance, Shanghai Business School, No. 2271 West Zhong Shan Road, Shanghai 200235, China; ^3^School of Business, Sanda University, No. 2727 Jinhai Road, Shanghai 201209, China; ^4^School of International Economics and Trade, Guangxi University of Foreign Languages, No. 19 Wuhe Avenue, Qingxiu District, Nanning, Guangxi 530222, China

## Abstract

Online healthcare platforms serve not just as a medical knowledge-sharing community but also bring about effective interactions between professional physicians and patients. However, it is unclear whether online technology adoption affects such interactions in the same way between traditional Chinese medicine and modern medical departments. By utilizing a large sample of online doctor-patient interaction information from 168,870 doctor-specific interactive webpages recorded in a famous Chinese online healthcare community, this paper studies the differences between 17,513 traditional medicine doctor homepages and 151,357 others from more than 100 different specialty areas. Our chosen platform is representative since it covers about 800,000 physicians working at over 10,000 hospitals across all major provincial regions in China. We document that online medical service users tend to accept and use online health care services. However, patients seeing Chinese medicine doctors exhibit the following unique characteristics. They still prefer choosing doctors according to third-party information and may be reluctant to pay for the current online service price level. This problem is hard to overcome by the platform in the short run. Patients need a long-term process to adapt to the upgraded medical environment gradually. Therefore, establishing a personalized doctor recommendation system has become the most urgent demand presently.

## 1. Introduction

As China chooses the urban-centered development route in the early stages of its economic and social growth plan, healthcare services and medical treatments, like other core resources, have experienced large urban-rural differences in distribution. Because of the dearth of quality hospitals, people in need of such care and consulting services are facing a series of problems with insufficient doctor-patient interaction, especially in the field of complementary, traditional, and alternative medicine [1]. In particular, patients with difficult, complicated, and miscellaneous diseases in remote areas often need to move around among multiple cities to finally obtain appropriate medical assistance. It is common for patients to transfer from county-level hospitals to municipal ones and then to provincial hospitals. Besides, patients still face the problem of long queues for registration [2]. Although the application of the hospital's online appointment system has improved the doctor-patient relationship, patients' trust in doctors and hospitals still needs more open and transparent information system support [3].

With the development of Internet technologies, the online health care (OHC) community represented by the Good Doctor (“Hao Daifu” in Chinese) and the Dr. Clove (“Dingxiang Yisheng” in Chinese) platform has gradually emerged as a rising medical force in China [4], which significantly eases the pressure on the entire medical system and effectively shortens the gap between urban and rural medical standards [5]. With the development of information and communication technology, many scholars have studied the impact of the Internet on the doctor-patient relationship and health outcomes [6]. Patients' acceptance of and willingness to pay for OHC services are both undoubtedly critical issues for the development of OHC platforms, especially when OHC appears to be a phenomenal application. Although OHC has become a hot topic in recent years, especially after the COVID-19 epidemic, and papers on the development of OHC have emerged in a huge stream, the question of whether complementary and traditional medicine are affected similarly in comparison to mainstream and modern medicine remains unanswered. This paper attempts to shed light on it.

Given the boom in China's online healthcare services, many people try to visit OHC websites for healthcare information and consultations [7]. The online medical care community can be defined as a medical career, which means attaining successful medical information exchange through remote electronic communication and providing treatment suggestions in conjunction with supporting and improving patients' clinical conditions [4]. Using texts and images, doctors can communicate their pieces of medical advice to patients and get feedback, which differs to a large extent from traditional face-to-face diagnosis and treatment. To meet complex needs, one may categorize OHCs into those having cure-oriented (i.e., functional) goals and those pursuing care-oriented (i.e., emotional) values [8]. The discussion presented in this paper mainly positions the analysis of comparing online traditional versus modern medical services in a cure-oriented OHC.

In regard to seeing a doctor online, there are essentially three types of doctor-patient interactive modes on the Internet that have been studied in the extant literature. The first involves making an appointment online and then meeting offline [9]. The second relates to selecting doctors online and consulting online too [10]. The third mode is mixed, including the online selection of and consultation with doctors, then switching to offline examinations if needed [11]. However, all the above three processes emphasize the same thing: choosing the right doctor. This has to be done either by word of mouth or by browsing each interested doctor's online web page.

As one kind of expert service, physicians' service inevitably leads to information asymmetry between doctors and patients [12]. In addition, because services are intangible, indivisible, and heterogeneous [13], it is hard for patients to judge the quality of the service they receive because doctors usually share much more professional knowledge than patients do [14]. In the highly centralized and miscellaneous OHCs, without mentioning the supervision of the platforms, patients actually take more risk and responsibility in how to choose the quality of doctor's service [10]. However, compared with offline hospitals, OHCs provide more useful information besides names and clinic titles [15], and users can even read the comments [10]. They found that patient-generated information, such as feedback, reviews, and ratings, will significantly affect patients' choices during their search stage. Besides, at the decision stage, system-generated information, such as contribution, grade, and popularity, will play an important role along with patient-generated information, which effectively weakens information asymmetry. Cao et al. [16] also prove that patients' information processing affects their online health consulting intentions in the same way. Lu and Wu [17] found that word-of-mouth information about physicians reflects the physicians' service quality and exerts a positive effect on persuading patients to make appointments. They also claim that patients with severe illnesses are more motivated to seek high-quality medical services.

Our study is complementary to the strand of literature on utilizing online technology to deal with the challenges faced by promoting alternative and traditional medicine. On the one hand, several recent studies combine various aspects of Traditional Chinese Medicine (TCM) with new developments in technology, including the Internet tool. For example, Han et al. [18] considered the Internet hospital as a telehealth model in China and applied a systematic search and content analysis to explore its patterns. Wang et al. [19] focus on TCM and study its traceability system based on a lightweight blockchain. Zhang et al. [20] took a relevant but different perspective by evaluating the effectiveness of online courses on TCM for international students during the COVID-19 epidemic period.

On the other hand, quite a few Chinese studies also provided insights. Based on the survey data, Ding et al. [21] constructed the evaluation index system of the information service quality of Internet Chinese medicine hospitals by combining the main characteristics of the information service quality of domestic Internet Chinese medicine hospitals. Wang [22] analyzed the current status of TCM intelligent technology, Internet service, healthcare, and community hospitals, and discussed the new development model of community hospitals based on Internet and TCM intelligence, to better meet the health service needs of the public. As the COVID-19 epidemic era has significantly boosted the development of “Internet+” Chinese medicine medical treatment, a growing strand of literature can study various TCM topics under the background of “Internet+”. For example, Cheng [23] explored how to optimize the effect of Chinese medicine in integrated family hospital management and the possible applications based on “Internet+” tools. Ma [24] proposed an “Internet+” Chinese medicine health management model to promote nursing treatment for elderly hypertensive patients. Cai et al. [25] argued that Internet medical technology can improve the treatment efficiency and accuracy of Chinese medicine acupuncturists by innovating the acupuncture treatment mode, optimizing the acupuncture treatment process, and improving patient health management. Sun and Ge [26] used the SWOT approach to analyze “Internet + TCM nursing service,” and discussed how to promote the innovation of TCM nursing health services and the associated risk issues. Tian and Gao [27] summarized the current situation of “Internet+” TCM medical development in China. Through analyzing the bottlenecks faced by the Internet TCM model, the authors make several suggestions, such as further enhancing the technology, educating personnel, and improving the medical insurance policy and legal regulation. Therefore, our comparative study of the doctor-patient online communication between TCM departments and others can help deepen the understanding of this “Internet+” TCM notion, providing supportive evidence for bettering the education of TCM and cultivating the public's online treatment habit in Chinese and alternative medicine.

When OHC became increasingly popular, related laws and regulations were gradually improved, and corresponding review mechanisms were also developed by well-known platforms. OHCs are gradually eliminating the barrier between patients and doctors. For example, the research results of Beaudoin and Tao [28] showed that the Internet can help cancer patients face disease, receive treatment, and seek support. Patients now not only have higher autonomy in choosing doctors but can also refer to more reliable information. Doctors with the ability and spare time can also create higher personal and social values while serving more patients [4]. Therefore, with the emergence of OHC, doctors can provide better and more personalized health care, and patients can get better health outcomes [29]. Although OHC is developing in full swing, its popularity, availability, and current acceptance, a.k.a., whether patients are willing to receive this kind of service and pay for it, still need further investigation. Based on this, we put forward the following three questions.

First, the telemedicine portal provides a wealth of personal information about doctors, but before the user's browsing behavior occurs, the only information source available on the portal is the brief information on the doctor's business card. Can this information fully induce the user's browsing behavior? In other words, is the user's browsing behavior purposeful or random? Based on this, we can judge the direction and extent of the actual effectiveness of Internet medical care. Second, the user's behavior in paying for the OHC platform service can fully show the patient group's preference for a certain type of doctor and enables us to make suggestions for better training of telemedicine service personnel. Third, there are great differences in the ways of interrogation and treatment between traditional Chinese medicine and modern medicine. Modern medicine often relies on high-end medical devices to assist in the completion of interrogation and treatment, while traditional Chinese medicine can often complete the treatment through the intuitive observation of the physiological characteristics of patients. In terms of drug treatment, traditional Chinese medicine should also be more convenient than modern medicine. Traditional Chinese medicine can often be easily purchased in local hospitals. Therefore, is there any difference in the convenience that telemedicine brings to the field of traditional and alternative medicine? In this regard, we start the analysis with the responses of Internet OHC user behavior to doctor information presented and practices delivered online.

## 2. Data and Empirical Method

### 2.1. Data Sources

Among the emerging Chinese OHC platforms, we collect data from the most representative Good Doctor Internet hospitals. This platform was established in 2006 with the brand feature of “user-friendly”. As of June 2022, the platform includes 890,286 doctors from 10,096 hospitals across China. In this study, we used the public data of this platform, including doctors' resumes and their evaluations by Internet patients, since they started an online service, which provided a rich set of data available for our research. In addition, as the most recognized and representative OHC platform in China, the Good Doctor website is chosen by many scholars to study various topics around the theme of OHC, which further reflects the reliability and availability of its data. All variables used in our research are listed and defined in Table 1.

**Figure 1:**
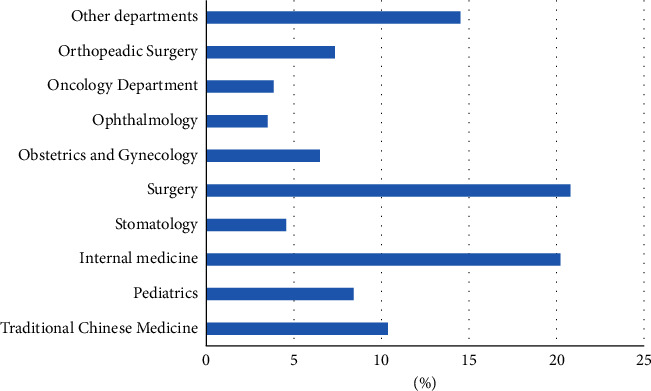
Proportion of doctors belonging to each hospital department.

**Figure 2:**
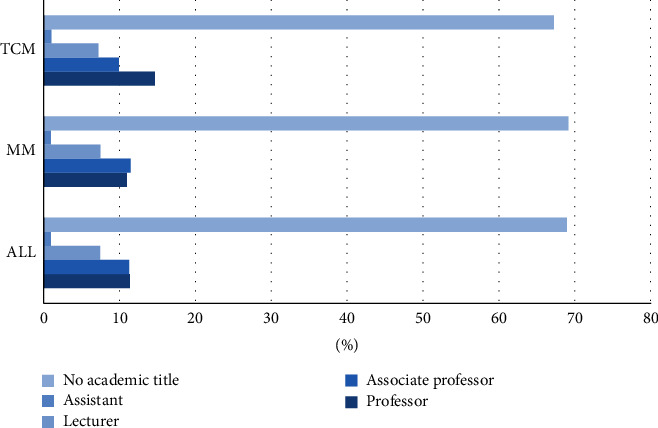
Proportion of doctors associated with each academic title in the TCM vs. MM department.

**Figure 3:**
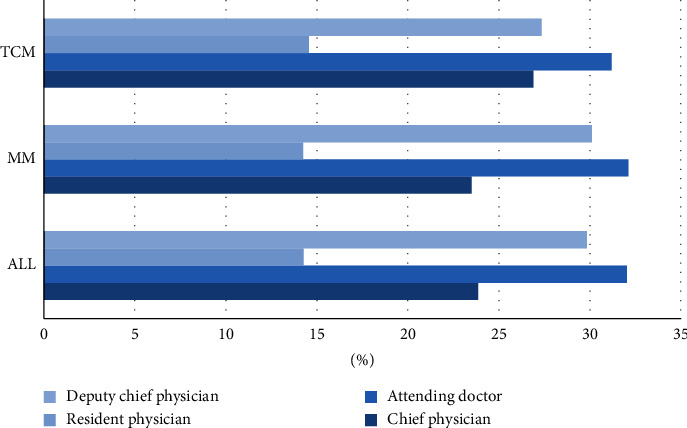
Proportion of doctors associated with each clinic title in the TCM vs. MM department. *Note*. The other four clinic titles together account for only a small proportion, hence not listed in the figure.

The data were collected through web scraping between June 31^st^, 2019 and August 2^nd^, 2019, including three dimensions of records. First, the basic information on the doctor's business card, including the doctor's name, academic title, clinic title, employer, hospital, and department. Second, personal introduction, including their specialization and personal profile. Third, personal achievements, including page views, number of published articles, number of patients, number of reviews, number of thank letters and gifts received, search heat, recommended star, and the year of starting service. Given the availability of data, we used the data of 168,870 doctors who went online from 2008 to 2019. These doctors come from ten main departments, as shown in Figure 1. Among them, doctors from TCM departments account for 10.4% and doctors from modern medicine (MM) departments account for 89.6% of all doctors in our sample. No significant differences are found for the two categories regarding the percentages holding advanced clinic titles and academic titles, as shown in Figures 2 and 3. The online doctor resources on the Good Doctor website are quite high quality. 11.4% of the doctors have the academic title of professor, and 23.9% of the doctors have the practical title of chief physician.

### 2.2. Variable Construction

To compare the differences in online patient behaviors concerning the TCM department and other hospital departments, we employ two commonly used proxies suggested by scholars specializing in Chinese OHC research as our dependent variables. They are doctor web page visits and the number of medical service orders. In particular, we follow Yu et al.'s [30] research on the causal effect of honorary titles on the volume of services provided by physicians and Li et al.'s [11] research on patients' decisions to switch from online to offline medical services. First, the volume of OHC users browsing a web page of a doctor, a.k.a. pageviews, is the result generated after patients decide to choose OHC and conduct a series of spontaneous searches. In essence, it is a direct reflection of the website's communication power and recognition. However, the internal driving force of users' browsing behavior should be to seek famous doctors rather than famous websites. It is also reasonable to believe that patients will check multiple platforms. Therefore, based on the data from the most representative and popular OHC platform, we think page view can show the impact and acceptance of OHC. Since one of the purposes of our research is to study the logic of patients' browsing behavior, it is understandable to use this simple but powerful indicator.

Turning to our second proxy of the volume of medical service orders, users placing orders and making payments will clearly reflect their preference for doctors as a direct result of browsing. Theoretically speaking, patients mainly use three information sources when selecting doctors: online reviews, comments, or feedback, family and friend recommendations; and doctor referrals [31]. The decision of patients to select doctors, that is, their order-placing behavior, is not only determined by the information provided by the platform but also by factors outside the system. However, the doctor's profile and personal achievement information provided by the platform, as well as the scoring of doctors' popularity and recommendations, can explain the uncertain factors caused by the differences in users' reference checks to a certain extent. Therefore, using the criteria provided by the platform to study the behavior of placing orders, we should be able to accurately reflect the types of information that users value.

**Figure 4:**
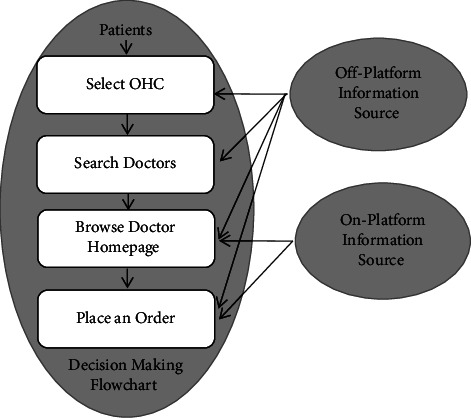
OHC user decision-making process.

Staying in line with the decision-making process in Figure 4 for patients utilizing the information received, a variety of public information types provided by the Good Doctor website are selected as explanatory variables and control variables. As we have discussed previously, the existing studies found that the information provided by OHCs plays a role in affecting the selection of doctors for OHC patients. According to Wu and Lu [32], in which they studied patient satisfaction, these two authors mainly chose explanatory variables from five dimensions of medical services: namely, service price, service provision, service quality, service criterion, and local economic development. The logic of selecting these variables is to intuitively reflect the judgments made by users after receiving services. Similarly, our model mainly relies on the predictions made by users before placing an order. Our variable selection mimics their choice of variables. The only difference is that the price factor is dropped in this paper because the price is not a directly observable factor in the process of the user searching and browsing on our platform. We only incorporate the information sources that can be gradually learned by OHC users as described by our setup.

### 2.3. Empirical Specification

Based on the information received by users when they perform corresponding behaviors on the website, we divided the samples, simulated the decision process, and established models based on the three different evaluation measures to analyze the development status of OHC and the differences between telemedicine in the fields of traditional Chinese medicine and modern medicine.

We begin by presenting the linear empirical regression of a decision model of page view volume determination. The general process of searching for doctors on the Good Doctor website is to select the target city and target department, and finally, browse the doctor's brief information and decide whether to enter the details page. When deciding whether to browse details, users first receive information about the doctor's name, academic title, clinic title, hospital name, corresponding department, specialized field description, popularity, hot, and online consultation price. It is worth noting that 90% of the online doctors who are good doctors come from lower-ranked hospitals. Therefore, we will mark the hospital level according to whether the city where the hospital is located belongs to the second-tier group of large cities in China instead of using the rankings of the hospitals themselves. In addition, we use the length of the detailed description to reflect the doctor's skills. Academic titles, clinic titles, and their departments are transformed into a series of dummy variables represented by vectors (marked as bold in the following equation).

(1) $$\begin{array}{c} {\text{PAGEVIEW}}_{i}=\beta_{0}+\beta_{1}\cdot AT_{i}+\beta_{2}\cdot CT_{i}+\beta_{3}\cdot \mathrm{DEPARTMEN}T_{i}+\beta_{4}\times {\text{HOT}}_{i}+\beta_{5}\times {\text{DESC}}_{\text{LEN}}{\;}_{i}+\beta_{6}\times {\text{CITY}}_{i}+\varepsilon_{i}, \end{array}$$

where *i* denotes the doctor's homepage in our sample, and all variables are as defined in Table 1. Following the similar decision rules stated above, we construct the next empirical specification to describe the number of orders placed with the detailed information provided on a doctor's OHC page. Compared with the above model, the new variables added include the doctor's resume, number of published articles, historical page views, number of thank-you letters and gifts received, and recommendation stars. Due to the high causal relationship between views and orders, this paper does not use orders as the explanatory variable of page views but uses page views as the explanatory variable of orders. We believe that multiple views can increase the probability of placing an order for that doctor's online service provided.

(2) $$\begin{array}{c} {\text{ORDER}}_{i}=\beta_{0}+\beta_{1}\cdot AT_{i}+\beta_{2}\cdot CT_{i}+\beta_{3}\cdot \mathrm{DEPARTMEN}T_{i}+\beta_{4}\times {\text{HOT}}_{i}+\beta_{5}\times {\text{CITY}}_{i}+\beta_{6}\times {\text{DESC}}_{\text{LEN}}{\;}_{i}+\beta_{7}\times {\text{ARTICAL}}_{i}+\beta_{8}\times {\text{REVIEW}}_{i}+\beta_{9}\times {\text{THANK}}_{i}+\beta_{10}\times {\text{GIFT}}_{i}+\beta_{11}\times {\text{INTRO}}_{\text{LEN}}{\;}_{i}+\beta_{12}\times {\text{STARTYEAR}}_{i}+\beta_{13}\times {\text{PAGEVIEW}}_{i}+\beta_{14}\times {\text{STAR}}_{i}+\varepsilon_{i}. \end{array}$$

## 3. Results and Discussion

### 3.1. Preliminary Inspection

We first present the descriptive statistics of our main variables in Table 2 across different samples (i.e., the full sample, the TCM observations, and all other non-TCM observations) and then compute the pairwise correlation coefficients between them in the whole sample. As can be seen from Table 2, doctors from the TCM and those from other departments on average provide about the same number of paid online medical services, while doctors in the TCM department attract more than 20% higher attention than non-TCM doctors as measured by the mean number of page views. We find no significant and consistent difference for all other variables across the two samples. Given that TCM doctors only account for about 10% of all doctors in our sample, the above observation crudely reveals that doctor-patient interactions can be more active for traditional medicine consultations in an Internet setup. Nevertheless, for all variables, the maximum values of the sample of modern hospital departments are noticeably higher than the corresponding values of the TCM sample. The same comparison relationship is also true for the values of variable standard deviations. In addition, almost all of our variables are significantly correlated with each other. Except for the year of being online, all explanatory variables have a positive association with both the proxies of our dependent variables, namely, page view and order. These facts imply that there exists a high degree of fluctuation in the potential explanatory variables within and across subsamples that allows us to identify the potential determinants of the higher number of web page visits to TCM doctors in the Internet hospital community. As a final check, Table 3 presents the pairwise correlation matrix. On an average, our concerned variables are significantly correlated with a moderate level of correlation coefficient magnitude, except for the high associations among the three proxies of review, thank-you, and gift for physicians' online professional performance.

### 3.2. Comparative Regression Analysis

We first regress web page visits, a.k.a. page views, on doctor characteristics for making comparisons between TCM and other doctors. The results in Table 4 show that the information on the doctor's visiting card cannot significantly explain the user's browsing behavior, but after adding more detailed information, especially the doctor's evaluation information, the browsing behavior can be fully explained. This implies that the generation of page views may not be random and groundless, and users may have certain intrinsic motivations and clear directions when searching for doctors. Given that the main information channels for Chinese users to select doctors are online comments and doctor recommendations, as well as family and friend recommendations, patients will use different information sources when selecting different departments. For example, when selecting traditional Chinese medicine, family and friend recommendations are the most dependent on patients. This finding explains the characteristics of browsing behavior to a certain extent. However, this behavioral logic also exposes the limited subjective initiative and limitations of OHC. That is, patients can learn about their condition through online doctors, but the excessive celebrity effect is likely to reduce the efficiency of medical resource redistribution, which may lead to a more serious occupation of cutting-edge doctor resources while middle- and low-end doctor resources are unoccupied to excess.

However, the behavior of patients' final choices can be significantly explained by the detailed information provided by doctors, especially by evaluative factors. We then replace the previous page view dependent variable with the order volume proxy and incorporate additional potential controls. Wang et al. [33] also found that doctors' online reputations will affect consumers' behavior in consultation. From the perspective of feedback, Yang and Zhang [34] discovered similar conclusions, and they further documented that the feedback of paid services has more reference value for patients. This implies that there exists a brand effect in the TCM field of OHC. Patients will try to find and choose doctors with good medical skills and reputations through various channels. Another crucial factor is the unintentional promotion of the website. Doctors with a large number of orders completed will be ranked at the top of the list.  Table 5 summarizes our findings by comparing the order number determination regressions in the TCM sample and the sample of non-TCM departments. It seems that online patients and OHC evaluations play a much larger role in increasing the number of TCM orders than other hospital departments. However, doctor-specific characteristics seem to possess no such power.

When comparing TCM with other MM departments, our finding is that when choosing modern medicine doctors on the Internet, users pay more attention to the degree of economic development of the doctor's residence city, while patients browsing TCM online content do not have such concerns. However, concerning placing medical service orders online, patients irrespective of TCM or MM collectively show great concern for regions. This phenomenon may be related to the better medical conditions of modern medicine in large cities. The academic titles of doctors in MM departments matter a lot to patients. However, titles seem to play a lesser role for TCM departments. In terms of goodness of fit, the browsing volume model with more regressors can explain the variations of the dependent variable much better for observations in the TCM department sample than in other departments. Besides, the order volume model also worked better in explaining the TCM department, which indicates that the online information about the TCM doctors is more efficient and the extent to which patients receive and process these pieces of information is also large. In contrast, the information of interest to Internet users of other medical departments has been, to some extent, hidden in the constant terms of our empirical setup.

One last aspect that merits note is that the TCM doctor with more online reviews or comments has a lower volume of web page browsing. However, the number of medical consulting orders placed is positively associated with the number of reviews. This might have something to do with the contents of the reviews. In the TCM field, it is very challenging to evaluate the effectiveness of a TCM treatment. As a result, both positive and negative reviews are more common for TCM doctors. Nevertheless, the number of orders and reviews should have a positive correlation in theory, regardless of good and bad comments. More orders will naturally lead to more views, but how many times a doctor's page has been viewed should be more related to the reputation of the doctor from a third-party perspective. We expect to dig deeper in this regard if detailed textual analysis can be performed on the comments.

### 3.3. Robustness Tests

Although the above empirical analysis preliminarily proves that the information provided by the website can help users make choices and well reflect the logic and preference of users' behavior, it is difficult to convincingly evaluate the acceptance of OHC and users' willingness to pay only by using simple quantitative variables. Moreover, either the number of page views or the number of orders placed for different doctors are not of the same degree of magnitude due to variations in their capability and popularity. Distortions in the estimate are likely to appear because of extreme values. For this reason, we use an alternative but relevant proxy to repeat our previous exercises as a robust test. In particular, we calculate the order conversion ratio to be the ratio of order volume to the number of page views. This is a standardized index, which can give us a unified evaluation standard to evaluate the ability and popularity of doctors with different attributes and more directly reflect the actual applicability of OHC. Compared with the single number of page views or orders, OCR contains more information, allowing us to analyze OHC acceptance and users' willingness to pay for OHC service in more dimensions. On the basis of the second model, we use OCR as the explained variable to establish the following model. Furthermore, because OCR is a positive number less than 1, we adopt the Probit model to analyze our results in addition to the baseline ordinary least squares (OLS) specification. The corresponding results are summarized in Table 6. It is demonstrated that the main findings of this article stay almost unchanged with alternative variable construction methods and alternative empirical estimation setups.

(3) $$\begin{array}{c} {\text{OCR}}_{i}=\beta_{0}+\beta_{1}\cdot AT_{i}+\beta_{2}\cdot CT_{i}+\beta_{3}\cdot \mathrm{DEPARTMEN}T_{i}+\beta_{4}\times {\text{HOT}}_{i}+\beta_{5}\times {\text{CITY}}_{i}+\beta_{6}\times {\text{DESC}}_{\text{LEN}}{\;}_{i}+\beta_{7}\times {\text{ARTICAL}}_{i}+\beta_{8}\times {\text{REVIEW}}_{i}+\beta_{9}\times {\text{THANK}}_{i}+\beta_{10}\times {\text{GIFT}}_{i}+\beta_{11}\times {\text{INTRO}}_{\text{LEN}}{\;}_{i}+\beta_{12}\times {\text{STARTYEAR}}_{i}+\beta_{13}\times \text{STAR}+\varepsilon_{i}. \end{array}$$

When we focus on OCR, the interpretation effect of the information provided by the website is greatly reduced, which may be caused by individual differences in patient behavior. Because the number of views generated by different patients is random, not all browsing behaviors will eventually lead to order placement. However, the previous results are still valid, and the evaluation information about doctors is still the most important factor for users. The significant constant term and low fitting coefficient also show that the ordering behavior and browsing behavior are not unified in most cases, and the users' choice is disturbed by strong external factors.

By comparing traditional Chinese medicine with modern medicine, we find that “whether the hospital is located in large cities” is an irrelevant variable for the order conversion ratio of traditional Chinese medicine departments, but it is a significant variable for modern medical departments, which indicates that OHC gives full play to the resources for scheduling medical resources and promotes the redistribution of high-end medical resources to underdeveloped areas. At the same time, nonacademic TCM doctors have been exposed and visited more. In addition, in the process of browsing and placing orders, people who choose traditional Chinese medicine departments do not care about the academic and professional titles of doctors, but people who choose modern medicine will consider the academic title of doctors. This may also be determined by the traditional differences between TCM and modern medicine. Unlike the findings from the two previous models, the OCR determination specification performs less well in TCM than in other departments. Considering that we have far more than usual browsing and query data in TCM, we believe that this result is caused by the presence of excessive invalid browsing in TCM.

## 4. Further Discussion

According to the Department of Health and Human Services, more than 60% of medical institutions and 40% to 50% of hospitals in the United States already use some form of telemedicine [35]. It is worth mentioning that telehealth in the United States is not in the state of being relatively separated from the public medical system like in China, but is attached to the hospital where the doctor works, which is more similar to the expanded version of the online appointment system. Compared with China's OHC platforms, it lacks integration, but the regulatory responsibilities are mainly undertaken by the American Telemedicine Association and other industry groups [36], while not by private companies and general governance departments. Moreover, the acceptance of telemedicine by Americans is much higher than that of China. According to a report by Kaiser Permanente of Northern California, the telehealth service has exceeded in-person visits by Advisory Board [37]. It is not difficult to conclude that China's OHCs are still immature in promoting telemedicine services, and there is still a long way to go. In this regard, we propose the following three suggestions.

The latest research points out that the professional ability, integrity, online reputation, and patient group size of psychological counselors have a significant positive impact on patient initial trust. Initial trust and the efforts of psychologists have a significant impact on sustained trust [38]. Whether this conclusion can be further extended to other departments still needs more experience. How to further promote telemedicine and help patients trust remote doctors? Providing more reliable information sources may be a worthwhile attempt. Therefore, a personalized doctor recommendation system is most needed at present. Secondly, the effective narrative turn of doctor-patient communication can be very useful to strengthen trust, improve the doctor-patient relationship, and increase the efficiency of treatment [39]. In the current narrative transformation of doctor-patient communication, in addition to necessary treatment and care for patients, more empathy and communication should be given in a narrative way [40]. The development of telemedicine also needs to consider this point of view. Last but not least, the platform should take more responsibility to avoid profit-making, false publicity, and promotion. This problem is much more serious in online traditional Chinese medicine departments. Although online TCM is theoretically more in line with the OHC diagnosis method, Chinese patients' long-standing habit of choosing doctors through offline interactions makes online TCM diagnosis results more of a reference. However, these facts also imply that TCM has a high degree of recognition in China and has a large room for exploring its potential applications in OHCs.

## 5. Concluding Remarks and Extensible Directions

Through the above analysis, it is easy to see that the current information provided by OHC is not enough to support or encourage patients to visit their doctors online, and patients often use third-party information actively or passively to make decisions. However, we are glad to see that users are happy to use the information on the OHC platform as a reference for medical treatment, especially when it is the most frequently browsed department, as represented by traditional Chinese medicine. Among these limited information sources, the factors that directly reflect the ability of doctors, such as the various achievements of doctors on the platform, have become the most recognizable by patients. Interestingly, the academic title is more persuasive in modern medicine than the doctor's clinical title. In addition, whether the hospital where the doctor works is located in a larger city or not turns out not to be a factor to consider. This coincides with the idea that Internet hospitals decrease the importance of geographical factors. On the one hand, it shows that the medical resources of the platform are of sufficient quality to believe, weakening the user's preference for developed cities. On the other hand, it also shows that OHC has overcome the geographical factors to a certain extent and realized the remote redistribution of medical resources by using the Internet.

The three proxies all reflect the same problem, that is, although users are gradually trying to accept and use OHC, they still tend to choose doctors according to third-party information and may have a low willingness to pay for the current OHC price level. This problem is difficult to overcome by the platform in the short term. Patients need a long-term process to gradually adapt to the upgraded medical environment.

Regarding potential directions for future studies, our findings point out the following aspects. The volume of page views created by patients who want to see a doctor in TCM before selecting an OHC doctor is much larger than that of other departments, but the proportion of patients paying for diagnosis and treatment is much smaller than that of modern medical departments. The status quo of “view but no order” in TCM reflects the following potential problems. First of all, most of the patients who have completed the treatment of online TCM are attracted by the doctors' fame, but they will still actively check the cases and doctors for related information. Second, traditional Chinese medicine is the only alternative for patients. Although browsing occurs, they may eventually choose modern medicine. Third, some patients try to complete self-diagnosis through relevant comments because TCM often does not need CT, X-ray, and other precise examinations. Fourth, the price of online traditional Chinese medicine is relatively high for the low-income group, and patients will give up placing orders for OHC medical treatment. However, due to the purpose and scope of our research, we cannot make a specific conclusion. Future research may allow the analysis of a wider range of data to help identify and improve the development of Internet TCM.

## Figures and Tables

**Table 1:** Variable definition and description.

Variable	Definition
Page view	The number of visits to a doctor's home page at the good doctor online platform in our sample
Order	The number of patients that the doctor has helped after paid medical service orders have been placed online (cumulative counts since the doctor has started his or her online services)
OCR	Order conversion ratio, which is calculated as the ratio of the number of orders placed to the doctor to the view volume of that doctor's web page
Star	The degree of recommendation associated with the doctor rated by the good doctor website based on historical data (the rating is an integer from 0 to 5)
Hot	The degree of popularity assigned to the doctor by the Good Doctor website to a doctor based on historical data
Thank	The number of thank-you letters received by the doctor
Gift	The number of gifts received by the doctor
Article	The number of papers and articles published by the doctor
Review	Number of comments received by the doctor
Start year	The year that the doctor started online services on the platform
Desc_len	The length of the description of the doctor's field of expertise
Intro_len	The length of the introduction to the doctor's professional experience
City	Whether the hospital where the doctor works is located in the first-tier and second-tier cities (according to China business network, there are four first-tier cities (Beijing, Shanghai, Guangzhou, and Shenzhen) in China and their 30 second-tier cities are selected out of 337 Chinese cities at the prefecture level and above. The selection criteria encompass five dimensions based on commercial store data, user behavior data, etc.) in the corresponding province
Academic title (AT)	AT represents a series of dummy variables constructed based on the academic titles associated with the doctor, which include professor, associate professor, lecturer, teaching assistant, and none
Clinic title (CT)	CT represents a series of dummy variables constructed based on the clinic titles associated with the doctor, including chief physician, deputy chief physician, resident physician, attending doctor, attending examiner, attending physician, resident physician, and laboratory physician
Department	Department represents a series of dummy variables constructed based on hospital departments in which the doctor can be classified, including TCM, pediatrics, internal medicine, stomatology, surgery, obstetrics and gynecology, ophthalmology, oncology, orthopedic surgery, and other departments

**Table 2:** Descriptive statistics.

Variable	Whole sample: 168870				TCM sample: 17513				MM sample: 151357			
	Mean	Std. dev.	Min	Max	Mean	Std. dev.	Min	Max	Mean	Std. dev.	Min	Max
Page view	306001	1712036	671	167000000	411237.50	2786098	892.00	167000000	293824.50	1539700	671	138000000
Order	344.52	1334.86	0.00	73374.00	333.38	1472.95	0.00	59248.00	345.81	1317.95	0.00	73374.00
OCR	0.00	0.00	0.00	0.52	0.00	0.00	0.00	0.03	0.00	0.00	0.00	0.52
Star	0.16	0.64	0.00	5.00	0.09	0.47	0.00	5.00	0.17	0.65	0.00	5.00
Hot	3.61	0.31	0.00	5.00	3.75	0.28	0.00	5.00	3.60	0.31	0.00	5.00
Thank	8.18	33.90	0.00	1632.00	5.04	20.20	0.00	559.00	8.55	35.12	0.00	1632.00
Gift	20.86	115.54	0.00	9362.00	13.54	84.76	0.00	3353.00	21.71	118.56	0.00	9362.00
Article	7.32	28.15	0.00	313.00	15.24	51.05	0.00	313.00	6.41	21.77	0.00	78.00
Review	19.46	71.16	0.00	3187.00	13.94	48.70	0.00	1332.00	20.10	73.29	0.00	3187.00
Startyear	2014.28	2.92	2008	2019	2013.77	3.01	2008	2018	2014.33	2.91	2008	2019
Desc_len	31.98	17.97	1.00	162.00	35.09	17.70	2.00	145.00	31.61	17.97	1.00	162.00
Intro_len	211.78	383.94	38.00	27056.00	214.32	294.04	38.00	9966.00	211.04	393.00	38.00	27056.00
City	0.50	0.50	0.00	1.00	0.35	0.48	0.00	1.00	0.52	0.50	0.00	1.00

*Note.* Other dummy variable series, including the academic title, clinic title, and department, are not shown to save space.

**Table 3:** Correlation matrix of main variables.

	Page view	Order	OCR	Star	Hot	Thank	Gift	Article	Review	Start year	Intro_len
Order	0.81^*∗∗∗*^										
OCR	0.01	0.12^*∗∗∗*^									
Star	0.23^*∗∗∗*^	0.44^*∗∗∗*^	0.33^*∗∗∗*^								
Hot	0.21^*∗∗∗*^	0.35^*∗∗∗*^	0.28^*∗∗∗*^	0.48^*∗∗∗*^							
Thank	0.47^*∗∗∗*^	0.68^*∗∗∗*^	0.14^*∗∗∗*^	0.55^*∗∗∗*^	0.47^*∗∗∗*^						
Gift	0.56^*∗∗∗*^	0.72^*∗∗∗*^	0.07^*∗∗∗*^	0.43^*∗∗∗*^	0.35^*∗∗∗*^	0.76^*∗∗∗*^					
Article	0.36^*∗∗∗*^	0.11^*∗∗∗*^	0.01^*∗*^	0.03^*∗∗∗*^	0.03^*∗∗∗*^	0.04^*∗∗∗*^	0.06^*∗∗∗*^				
Review	0.49^*∗∗∗*^	0.72^*∗∗∗*^	0.13^*∗∗∗*^	0.55^*∗∗∗*^	0.49^*∗∗∗*^	0.98^*∗∗∗*^	0.78^*∗∗∗*^	0.05^*∗∗∗*^			
Start year	−0.22^*∗∗∗*^	−0.21^*∗∗∗*^	0.19^*∗∗∗*^	−0.08^*∗∗∗*^	−0.20^*∗∗∗*^	−0.18^*∗∗∗*^	−0.15^*∗∗∗*^	−0.03^*∗∗∗*^	−0.21^*∗∗∗*^		
Intro_len	0.15^*∗∗∗*^	0.17^*∗∗∗*^	0.01^*∗∗∗*^	0.13^*∗∗∗*^	0.27^*∗∗∗*^	0.20^*∗∗∗*^	0.15^*∗∗∗*^	0.03^*∗∗∗*^	0.21^*∗∗∗*^	−0.26^*∗∗∗*^	
Desc_len	0.12^*∗∗∗*^	0.17^*∗∗∗*^	0.14^*∗∗∗*^	0.18^*∗∗∗*^	0.33^*∗∗∗*^	0.17^*∗∗∗*^	0.12^*∗∗∗*^	0.02^*∗∗∗*^	0.18^*∗∗∗*^	−0.22^*∗∗∗*^	0.24^*∗∗∗*^

*Note.* ^*∗∗∗*^ and ^*∗*^ represent significance at the 1% and 10% levels, respectively.

**Table 4:** The effects on page views for doctors in the TCM and MM departments.

Variables	(1) All	(2) All	(3) TCM	(4) MM
	Page view	Page view	Page view	Page view
Desc_len	3.88^*∗∗∗*^	1.50^*∗∗∗*^	1.36^*∗*^	1.07^*∗∗∗*^
	(0.24)	(0.19)	(0.81)	(0.18)
Star	541.29^*∗∗∗*^	−123.57^*∗∗∗*^	−266.04^*∗∗∗*^	−136.84^*∗∗∗*^
	(6.45)	(6.18)	(35.13)	(5.86)
City	104.45^*∗∗∗*^	17.75^*∗∗∗*^	−42.01	29.77^*∗∗∗*^
	(8.20)	(6.56)	(27.35)	(6.39)
Hot		−257.60^*∗∗∗*^	−417.36^*∗∗∗*^	−221.01^*∗∗∗*^
		(14.15)	(58.82)	(13.81)
Thank-you		−12.04^*∗∗∗*^	23.51^*∗∗∗*^	−11.37^*∗∗∗*^
		(0.45)	(2.76)	(0.43)
Gift		6.26^*∗∗∗*^	15.75^*∗∗∗*^	5.48^*∗∗∗*^
		(0.04)	(0.27)	(0.04)
Article		2.56^*∗∗∗*^	2.32^*∗∗∗*^	5.73^*∗∗∗*^
		(0.01)	(0.02)	(0.06)
Review		9.50^*∗∗∗*^	−7.05^*∗∗∗*^	9.34^*∗∗∗*^
		(0.23)	(1.15)	(0.22)
Start year		−62.64^*∗∗∗*^	−66.14^*∗∗∗*^	−59.05^*∗∗∗*^
		(1.24)	(4.98)	(1.21)
Intro_len		0.13^*∗∗∗*^	0.38^*∗∗∗*^	0.10^*∗∗∗*^
		(0.01)	(0.05)	(8.48)
Academic title dummies	Yes†	Yes†	Yes	Yes†
Clinic title dummies	Yes	Yes	Yes	Yes
Department dummies	Yes†	Yes†	Yes	Yes†
Constant	252.94	127309.8^*∗∗∗*^	134057.7^*∗∗∗*^	119594.3^*∗∗∗*^
	(1164.83)	(2643.47)	(10156.98)	(2443.94)
Observations	168870	168870	17513	151357
R-squared	0.075	0.452	0.621	0.428

*Note.* Page view is measured in thousands of web page visits. Standard errors are in parentheses. ^*∗∗∗*^ and ^*∗*^ represent significance at the 1% and 10% levels, respectively. For all AT, CT, and department dummy variables included in the regression, we mark † only when more than half of the dummy coefficients turn out to be significant.

**Table 5:** The effects on the volume of doctor service orders in the TCM and MM departments.

Variables	(1) All	(2) TCM	(3) MM
	Order	Order	Order
Desc_len	1.13^*∗∗∗*^	0.72^*∗∗∗*^	1.11^*∗∗∗*^
	(0.11)	(0.24)	(0.13)
Star	193.91^*∗∗∗*^	289.00^*∗∗∗*^	189.62^*∗∗∗*^
	(2.72)	(10.39)	(2.80)
City	−39.14^*∗∗∗*^	−23.63^*∗∗∗*^	−39.52^*∗∗∗*^
	(2.73)	(7.91)	(2.90)
Hot	−41.82^*∗∗∗*^	58.41^*∗∗∗*^	−50.10^*∗∗∗*^
	(6.21)	(17.60)	(6.74)
Thank-you	−15.01^*∗∗∗*^	−15.42^*∗∗∗*^	−15.43^*∗∗∗*^
	(0.20)	(0.80)	(0.24)
Gift	1.22^*∗∗∗*^	0.34^*∗∗∗*^	1.30^*∗∗∗*^
	(0.01)	(0.10)	(0.01)
Article	−0.78^*∗∗∗*^	−0.91^*∗∗∗*^	−0.29^*∗∗∗*^
	(0.01)	(0.01)	(0.01)
Review	11.96^*∗∗∗*^	11.07^*∗∗∗*^	12.17^*∗∗∗*^
	(0.11)	(0.32)	(0.14)
Start year	6.44^*∗∗∗*^	6.60^*∗∗∗*^	6.60^*∗∗∗*^
	(0.53)	(1.51)	(0.64)
Intro_len	−0.01^*∗∗∗*^	−0.09^*∗∗∗*^	−0.03^*∗∗∗*^
	(0.01)	(0.01)	(0.01)
Page view	0.01^*∗∗∗*^	0.01^*∗∗∗*^	0.01^*∗∗∗*^
	(0.01)	(0.01)	(0.01)
Academic title dummies	Yes†	Yes	Yes†
Clinic title dummies	Yes	Yes	Yes
Department dummies	Yes†	Yes	Yes†
Constant	−12482.31^*∗∗∗*^	−13365.71^*∗∗∗*^	−13036.50^*∗∗∗*^
	(1137.22)	(2985.30)	(1150.92)
Observations	168870	17513	151357
R-squared	0.836	0.884	0.830

*Note.* Standard errors are in parentheses. ^*∗∗∗*^ and ^*∗∗*^ represent significance at the 1% and 5% levels, respectively. For all AT, CT, and department dummy variables included in the regression, we mark † only when more than half of the dummy coefficients turn out to be significant.

**Table 6:** Results of using alternative dependent variables and empirical specifications.

	(1) OLS all	(2) Probit all	(3) Probit TCM	(4) Probit MM
Variables	OCR	OCR	OCR	OCR
Desc_len	0.01^*∗∗∗*^	0.01^*∗∗∗*^	0.01^*∗∗∗*^	0.01^*∗∗∗*^
	(0.01)	(0.01)	(0.01)	(0.01)
Star	0.01^*∗∗∗*^	0.01^*∗∗∗*^	0.01^*∗∗∗*^	0.01^*∗∗∗*^
	(0.01)	(0.01)	(0.01)	(0.01)
City	−0.01^*∗∗∗*^	0.02^*∗*^	0.01	0.01^*∗∗*^
	(0.01)	(0.01)	(0.03)	(0.01)
Hot	0.01^*∗∗∗*^	0.50^*∗∗∗*^	0.49^*∗∗∗*^	0.50^*∗∗∗*^
	(0.01)	(0.02)	(0.07)	0.03
Thank-you	−0.01	0.19^*∗∗∗*^	0.14^*∗∗∗*^	0.19^*∗∗∗*^
	(0.01)	(0.01)	(0.02)	(0.01)
Gift	−0.01^*∗∗∗*^	0.82^*∗∗∗*^	0.76^*∗∗∗*^	0.84^*∗∗∗*^
	(0.01)	(0.01)	(0.03)	(0.01)
Article	−0.01	0.10^*∗∗∗*^	0.08^*∗∗∗*^	0.10^*∗∗∗*^
	(0.01)	(0.01)	(0.01)	(0.01)
Review	−0.01^*∗∗∗*^	0.04^*∗∗∗*^	0.01^*∗∗*^	0.04^*∗∗∗*^
	(0.01)	(0.01)	(0.01)	(0.00)
Start year	0.01^*∗∗∗*^	−0.04^*∗∗∗*^	−0.04^*∗∗∗*^	−0.04^*∗∗∗*^
	(0.01)	(0.01)	(0.01)	(0.01)
Intro_len	−0.01^*∗∗∗*^	−0.01^*∗∗∗*^	−0.01	−0.01^*∗∗∗*^
	(0.01)	(0.01)	(0.01)	(0.01)
Academic title dummies	Yes†	Yes†	Yes	Yes†
Clinic title dummies	Yes	Yes†	Yes	Yes
Department dummies	Yes†	Yes†	Yes	Yes†
Constant	−0.33^*∗∗∗*^	82.01^*∗∗∗*^	83.81^*∗∗∗*^	79.75^*∗∗∗*^
	(0.01)	(3.8)	(10.4)	(4.1)
Observations	168870	157459	16840	140619
R-squared	0.262	0.347	0.315	0.352

*Note.* Standard errors are in parentheses. ^*∗∗∗*^, ^*∗∗*^, and ^*∗*^ represent significance at the 1%, 5%, and 10% levels, respectively. For all AT, CT, and department dummy variables included in the regression, we mark † only when more than half of the dummy coefficients turn out to be significant.

## Data Availability

The dataset used for this study is compiled from the publicly-available physicians' webpages on the Good Doctor website.
